# American Dental Association

**Published:** 1867-08

**Authors:** 


### AMERICAN DENTAL ASSOCIATION.

This body held its seventh annual meeting in the City of Cincinnati, July 30th to August 3d, inclusive. It was regarded by those in attendance one of the best meetings ever held by this Association.

The time was most fully occupied, there being three sessions each day. There were in attendance one hundred and thirty-four members. There was a larger number of reports and papers than usual, presented, *read* and *discussed.* Most of the standing committees had reports, and there was quite a number of volunteer papers, all of which received due consideration.

There was a disposition to keep all vexed and disturbing questions in the background. The proceedings were harmonious throughout.

Those who were not present can scarcely realize how much they missed by being absent.

The Association concluded its sessions on the fifth day, amid almost unbounded enthusiasm and interest; and we can hope for no better thing for this Association, than that it may commence its next annual meeting just where, and with the same spirit it closed this year. The next meeting will be held at Niagara Falls.

